# Participation of Monocyte Subpopulations in Progression of Experimental Endotoxemia (EE) and Systemic Inflammation

**DOI:** 10.1155/2021/1762584

**Published:** 2021-02-12

**Authors:** Yaroslav V. Radzyukevich, Ninel I. Kosyakova, Isabella R. Prokhorenko

**Affiliations:** ^1^Hospital of Pushchino Scientific Center, Russian Academy of Sciences, Pushchino, 142290, Russia; ^2^Department of Molecular Biomedicine, Institute of Basic Biological Problems, Federal Research Center “Pushchino Scientific Center for Biological Research of the Russian Academy of Sciences”, Pushchino, 142290, Russia

## Abstract

Systemic inflammation plays a crucial role in formation of various pathological conditions, including sepsis, burns, and traumas. The main effector cells participating in progression of systemic inflammation response and sepsis are monocytes, which regulate both innate and acquired immunity via phagocytosis, synthesis of cytokines and chemokines, antigen presentation, and lymphocyte activation. Thus, the monocytes are considered as a link between innate and acquired immunity. The monocyte subpopulations taken into consideration in the study essentially determine the progression of systemic inflammation and could serve as targets for therapeutic intervention. The complexity of the analysis of pathophysiology of systemic inflammation lies in its high variability conditioned by individual peculiarities of the patients and inflammation progression specifications. To overcome these limitation, model of experimental endotoxemia (EE) is used. The results of EE, in turn, cannot be directly extrapolated on patients with the systemic inflammatory response. This review is dedicated to discussing the role of monocyte subpopulations in progression of systemic inflammation/sepsis and EE.

## 1. Introduction

Systemic inflammatory response syndrome (SIRS) is excessive protective reaction of the body on a damaging stress factor (infection, trauma, surgery, or acute inflammation) for localization and further elimination of endogenous or exogenous damaging agent. Systemic inflammation (SI) of suspected infectious origin is named sepsis [1]. The main challenge for patients with risk of SI and sepsis development is the impairment of synthesis of pro- and anti-inflammatory cytokines by the innate immune system cells. During the septic conditions, the innate immune system activated by PAMP and DAMP releases many proinflammatory cytokines during the process known as “cytokine storm,” which leads to the severe and robust inflammatory response. Besides that, excessive inflammatory reactions lead to cell and tissue damage leading to dysfunction of organs and even multiple organ failure. Another threat of such inflammation is the subsequent formation of immunosuppressive condition that facilitates the development of secondary infections [2]. In the current review, we regard SI as an important part of generalized nonregulated inflammatory response progression of infectious origin leading to development of sepsis.

Monocytes play the most important role in progression of inflammation and sepsis-induced immunosuppression. The population of monocytes is heterogenic, each subpopulation being characterized by specific functional features and playing its own role in the immune response. Studying the roles of the subtypes of monocytes in progression of SI and sepsis could help to develop effective targeted therapy against this severe disease [3].

The main problem in studying the mechanisms of sepsis progression is its multifactor nature and variability of the immune response between patients. As a result, a large group of patients is needed for clinical trials to determine the effects of the undertaken interventions. Even if this condition is satisfied, the positive results of preclinical studies often fail to be reproduced in clinical (phase III) trials [4].

The experimental model of human endotoxemia is used to overcome these limitations of extrapolation of the preclinical results onto clinical practice. Intravenous LPS administration to healthy volunteers causes short-term, tolerable, and controllable systemic inflammatory response imitating the primary inflammatory response observed in patients with SI [5]. Thus, EE is an example of translational research that helps to study the mechanisms of systemic inflammation and to estimate novel pharmacological interventions in humans in vivo [6]. EE can be regarded as an early step of the immune response to pathogen invasion [7, 8], whereas the long-term changes in immune cells are studied on whole blood of patients with clinically confirmed sepsis [9, 10].

The goal of the current review is comparison of the behaviour of monocyte subpopulations during EE and sepsis focusing on the possibilities and limitations of extrapolation of the results obtained on the model onto the real clinical conditions.

## 2. Monocytes

The monocytes regulate both innate and adaptive immune responses to pathogens and endogenous sterile stimuli via phagocytosis, release of reactive oxygen species, cytokines, and chemokines, recruiting of neutrophils, antigen presentation, and activation of lymphocytes [11]. The diversity of the performed functions is provided by heterogeneity of the population of these cells. Thus, the term “monocyte” can be attributed to the cells sharing common appearance but performing different functions [12].

The human monocytes are traditionally subdivided into three subsets based on different expression of a coreceptor to lipopolysaccharide (LPS), CD14, and CD16 receptor (Fc*γ*RIII) [13]. In 2010, the Nomenclature Committee of International Union of Immunological Societies [14] approved the conventional designations of three subpopulations of human monocytes: classical monocytes with high level of CD14 expression but not expressing CD16 (CD14++CD16-), intermediate monocytes expressing CD16 in addition to CD14 (CD14++CD16+), and nonclassical monocytes with virtually nondetectable CD14 and high expression of CD16 (CD14 + CD16++) [15]. In healthy humans, classical monocytes comprise around 85% of the total population of circulating monocytes, the intermediate ones comprise 5%, and the nonclassical ones are the remaining 10% [10].

It is conventionally believed that during human monocyte differentiation, the classical monocytes leave the bone marrow and are differentiated first into the intermediate monocytes and then form the nonclassical subpopulation in peripheral circulation in 2-7 days [16, 17]. The mature monocytes are present in circulation for 1.5 to 7.5 days, and after that, they either die or migrate to tissues where they differentiate into macrophages or dendritic cells [18].

Currently, the scientists try to detect novel markers allowing to decrease the effect of “human factor” and to determine the functions of one monocyte subpopulation or another more precisely. The following receptors are regarded as novel potential auxiliary specific surface markers for accurate determination of monocyte subpopulations in blood [19]:

- BLTR1, CD35, CD38, and CD89—markers of classical monocytes
- CD39, CD275, and CD305—markers of intermediate monocytes
- CD 29 and CD 132—markers of non-classical monocytes

**Figure 1:**
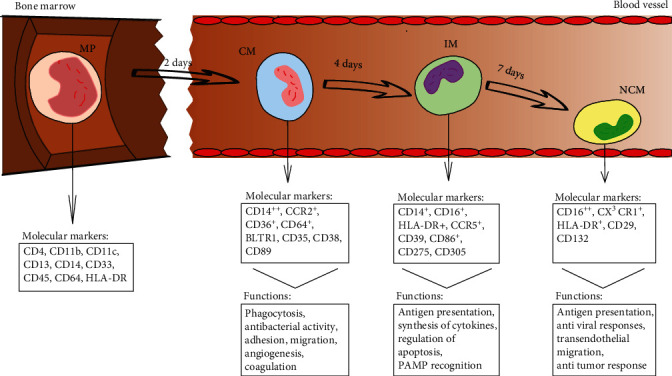
Human monocyte subsets in health. Human monocytes mature in the bone marrow and are subsequently released into the circulation as CD14++classical monocytes. Progressively, classical monocytes (CD14++CD16−) give rise to nonclassical monocytes (CD14dimCD16++) through an intermediate step of CD14 + CD16+ monocytes. MP: myelomonocytic progenitor; CM: classical monocytes; IM: intermediate monocytes; NCM: nonclassical monocytes.

Functional peculiarities of the subpopulations of monocytes are conditioned by various expression of molecules mediating recognition, phagocytosis, and antigen presentation (Figure 1).

## 3. Subtypes of Monocytes in Sepsis

The most important period in formation of septic condition is the first day from the invasion of the bacteria or their products into circulation, when the rapid inflammatory response is developed. Change of monocyte count, subpopulation ratio, and their functional characteristics plays an important role in this process. Nonetheless, there argues about whether the human endotoxemia is an appropriate model for studying sepsis and whether it is appropriate for studying the therapeutic strategies of treatment of this disease [20]. The important problem is the focus of the majority of modern studies, especially those related to the adaptive immune system, on the earliest phase after endotoxin invasion (the first 24 hours). The long-term effects in adaptive immunity are therefore out of account, only scarce information being available [21]. To check the applicability of ЕЕ as a model system for monitoring SI or sepsis progression, changes in monocyte subpopulations during endotoxemia and acute inflammation should be compared [22].

The immune paralysis conditioned by sepsis causes suppression of synthesis of cytokines TNF-*α*, IL-1*α*, IL-1*β*, IL-6, IL-10, and IL-12 by circulating monocytes [23] and the decreased expression of human leukocyte antigen HLA-DR on the surface of monocytes [24–26]. Loss of HLA-DR by circulating antigen-presenting cells (the monocytes being part of them) is related to alleviation of sensitivity to pathogenic microorganisms, and the lethality of patients during sepsis is related to inability of the monocytes to restore HLA-DR [27]. Despite deactivation, the monocytes are rapidly differentiating into subpopulations of dendritic cells (DC), which do normally induce either anergic T-cells, or proliferation of T-cells with regulatory potential. However, these monocyte-derived DCs are incapable of activating T-lymphocytes. Such alteration in the DC subpopulation results in decreased induction of cytokines [28]. Thus, the adaptive immunity is literally excluded from the response to pathogens, which significantly reduces the probability of benign outcome of the disease.

The significance of the role of monocytes in the development of sepsis and septic shock is shown by their effect on innate (alteration of cytokine expression) and adaptive (antigen presentation) immunity. This is exactly why mHLA-DR is the most studied marker of immune paralysis up to date [29].

## 4. Dynamics of Change of Absolute and Relative Count of Monocytes and Their Subpopulations during EE

**Figure 2:**
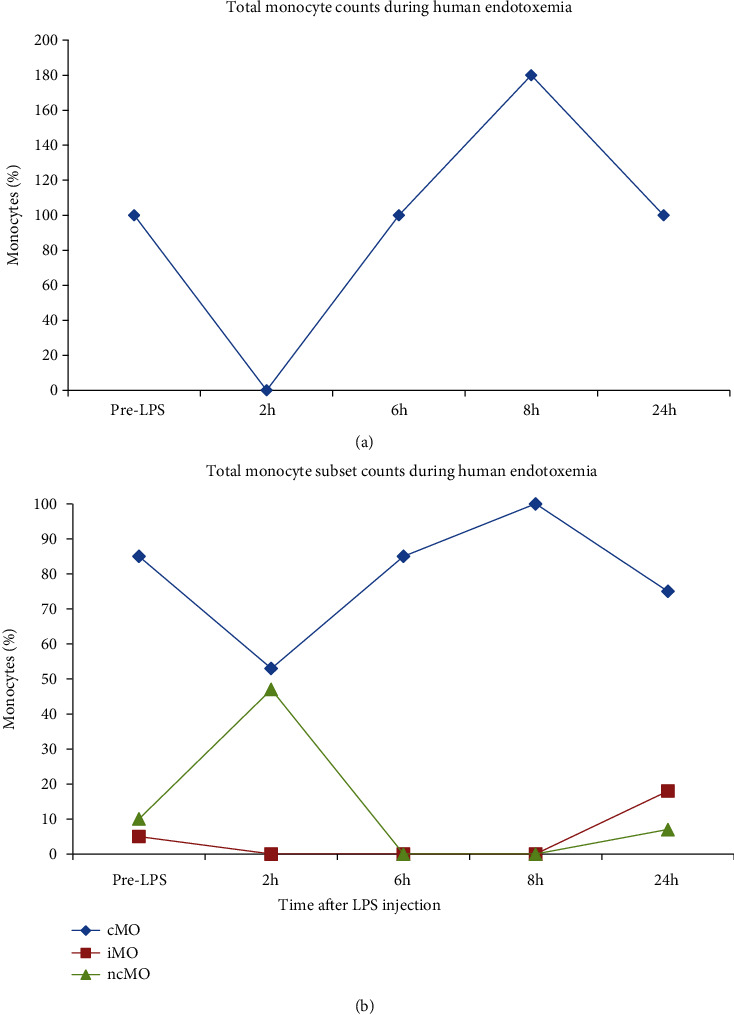
Monocytes in experimental endotoxemia. Total monocyte percentages (a) are decreased at 1–1.5 h after LPS injection, and cell count increases at 6–8 h after LPS injection. Monocyte subset percentages (b) show that classical monocytes (cMo) comprise 80–90% of all monocytes. cMo percentages follow those of total monocytes. Intermediate monocyte (iMo) and nonclassical monocyte (ncMo) percentages are decreased 1–8 h after LPS injection with a trend towards higher iMo percentage at 24 h after LPS injection [7, 8].

The total number of monocytes in blood decreases 1-1.5 hours after the i.v. administration of LPS and is recovered gradually in 4-6 hours. After eight hours, monocytosis, the significant increase of monocyte count in blood, is observed. The drastic loss of monocytes from blood circulation on early stages of inflammation could reflect the elevation of monocyte number in marginal pool near the vessel walls [30]. Increase of monocytes in 6-8 hours can reflect mobilization of marginal cells or compensatory release of the cells from bone marrow, where a large number of monocytes is located (Figure 2) [8].

Significant elevation of iMo count a day after the LPS administration when the level of two other subpopulations is normal forms the alteration of distribution in monocyte subpopulations in the direction of intermediate monocytes [31]. Moreover, the recent work by Rodriguez-Rosales and colleagues showed that this effect is maintained for at least 20 days during experimental endotoxemia [32].

Thus, the initial monocytopenia could be explained by adhesion of the activated cells to endothelium, which was confirmed in the work of Mukherjee and coworkers. They showed that the absolute and relative counts of monocyte subpopulations do not change during LPS activation of isolated blood [33].

Restoration of number of classical and nonclassical monocytes as well as increase of the quantity of intermediate monocytes could be related to both compensatory release of the cells from the bone marrow and sequential differentiation of the monocytes [7, 34].

Currently, there is a lack of clear understanding of the reasons for such a sharp change of count and percentage on monocyte subpopulations in blood after invasion of LPS. One can suppose that activation of cMo and iMo is determined by the need to eliminate bacteria and products of their decomposition entering the blood circulation, whereas exit from the vessels is caused by the necessity to protect tissues from the pathogen [8, 10]. In turn, significant elevation of iMo in 24 hours points on the essential cytokine response and activation of acquired immunity to protect the host [35].

## 5. Changes of the Quantity and Subpopulation Ratio of Monocytes during Sepsis

The dynamics of changes in the absolute and relative numbers of monocyte subpopulations in patients with sepsis varies in different studies. Mukherjee and coworkers observed a significant elevation of CD16+ monocyte quantity along with proportional decrease of cMo count [33]. Some authors showed that monocytosis is observed during sepsis [36], the number of classical and/or intermediate monocytes being elevated, which corresponds to the effects observed during EE [10, 37, 38]. ncMo did not display changes in number.

When bacterial sepsis is diagnosed, the ratio of subpopulations depends on presence of the bacteria in blood: the fraction of intermediate cells increases in cases of positive results of bacteriological cultures. After elimination of the bacteria, the ratio between subpopulations is restored [10]. On the contrary, Rodriguez-Rosales showed that the quantity of all the subpopulations of monocytes in circulation is noticeably decreased during sepsis. These changes remain for at least 9 days after the beginning of observation [32]. The observed effect has little coincidence with changes in the ratio of monocyte populations in experimental endotoxemia. Such differences in the results emphasize heterogeneity of sepsis, because the authors used different approaches to determination of septic condition of the patients. So, patients with different stages of the inflammatory response could fall into one experimental sample, contrary to the standardized experimental endotoxemia. Besides that, the accuracy of CD16+ isolation depends on the method of subpopulation gating and, finally, on the researcher himself [14, 32, 33].

## 6. Change of the Receptor Expression on Monocyte Subpopulations during EE and SI

Besides the quantitative changes in the monocyte population, the process of change of the expression of the surface receptor directly related to functional activity of the cells is of at least equal importance. The main receptors involved into response to LPS are TLR2, TLR4, CD14, CD16, CD11b/CD18, and HLA-DR [8, 33, 39].

Toll receptors (TLRs) are the receptors recognizing bacterial products and triggering the inflammatory reaction. There are 11 known receptors of this group, but the most interesting ones are TLR2 and TLR4, because they interact with gram-positive and gram-negative bacteria and their products, respectively. Mukherjee and colleagues showed that the expression of TLR2, or TLR4, and TLR5 is significantly expressed on the activated cMo and iMo, respectively [33]. Despite this study was carried out in vitro, which sets certain limitations on interpreting the results, elevation of expression of these receptors on monocytes was also found in vivo during SI [9, 37, 40].

CD11b is dimerized with CD18. It is a receptor necessary for adhesion and motion of the monocytes on the endothelial layer [41]. Moreover, CD11b is necessary as a coreceptor for LPS recognition. Inflammatory stimulation causes rapid elevation of the surface expression of CD11b in the intermediate monocyte subpopulation. This effect is observed in both EE and sepsis [40, 42]. Elevation of the CD11b expression in intermediate cells can give evidence on active adhesion of this subpopulation during inflammatory activation [7].

With the development of methods of determination of surface and intracellular receptors of the monocytes, the attention of the researchers focused on the novel markers of these cells. Decrease of coreceptor CD86 molecule points on the weakened antigen-presenting activity [8]. The low degree of activation can be related to migration of the activated cells into tissues, leaving the less activated cells in circulation. The analogous model, when only the cells with low activation degree remain in circulation, was proposed for neutrophils [43].

The monocytes of patients with sepsis show the decreased expression of CD86, HLA-DR, CCR2, and CD163 compared to the monocytes of healthy subjects [44]. The decrease of the expression of these receptors was also observed in EE [8; 44]. Such changes in receptor profile of monocytes signalize decrease of antigen-presenting, chemokine, and proinflammatory activities of these cells, respectively.

HLA-DR expressed by monocytes and other antigen-presenting cells is required for initiation of the adaptive immune response [45]. Underactivation by endotoxins, noticeable decrease of the HLA-DR level and percentage of HLA-DR positive cells in iMo and ncMo subpopulations is observed. HLA-DR is an especially interesting marker, because the decrease of its expression in monocytes was shown to correlate with development of sepsis after severe inflammation [46]. Alleviation of the HLA-DR expression during experimental endotoxemia can be explained by both losses of almost all the monocytes from circulation and loss of cells with high HLA-DR level, while the remaining antigen-presenting cells have lower HLA-DR level [8, 32].

Key differences between the characteristics of monocyte subpopulations during EE and SI are observed in the expression of CD14 and CD16 receptors. In the case of EE, the classical monocytes display decrease of the CD14 expression, whereas ncMo shows the alleviated CD16 expression 24 hours after the LPS administration [7, 39]. Alleviation of CD14 quantity can be attributed to its internalization for activation of the intracellular signaling pathway. Besides that, the soluble form of this receptor formed from membrane-anchored isoform actively participates in elimination of LPS from circulation. The decreased expression of CD16 was earlier explained by vanishing of CD16+ populations of Мо after LPS activation [39], but this proposal was disproved later [8]. How the decreased expression of CD16 affects the functional activity of ncMo remains unclear.

Contrary to the primary response on inflammatory stimulus during experimental endotoxemia, in sepsis, the density of the expression of CD14 receptor and IgG-receptors CD16 and CD64 increases, most significantly in the subpopulations of intermediate and nonclassical monocytes. Changes in the CD14 receptor expression depend on the etiology of sepsis, because they are observed in Gram(+)-sepsis. The patients with Gram(-)-sepsis do not demonstrate the changes in the surface expression of this receptor [47]. Simultaneous elevation of the expression of CD16 and TLR4 increase the activity of TRIF-pathway and synthesis of corresponding cytokines, whereas de novo expressed CD64 and CD16 promote enhanced phagocytosis of IgG-opsonized bacteria [10]. Elevation of the expression of CD16 and the relative level of CD16+ monocytes leads to formation of endotoxin tolerance [48].

Summarizing the changes in the expression of receptors on different subgroups of monocytes during EE and SI, one can suppose that the observed similarity in the receptor expression is conditioned by the need for rapid response to the stimulus focused on elimination of pathogen. Formation of endotoxin tolerance, which is directly related to CD14 and CD16 receptors, appears during repeated action of proinflammatory stimuli observed during systemic inflammation, which is absent after single LPS administration in case of EE. Currently, repeated or bolus infusion of LPS is used to obtain more reliable results and to study the mechanism of formation of tolerance [22, 49].

Comparison of changes of the expression of key receptors on monocyte subpopulations is presented in Table 1.

Table 1 shows that changes of expression of key surface receptors on the monocytes involved into the response to LPS in EE and monocytes participating in inflammatory response during SI are almost identical.

## 7. Cytokines and Chemokines

**Figure 3:**
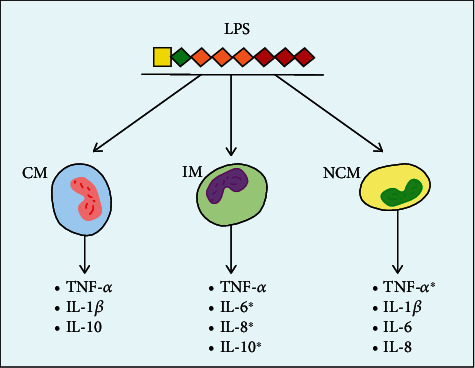
Expression of cytokines by subpopulations of monocytes after LPS activation. ^∗^The cytokines expressed mainly by this subpopulation of monocytes. MP: myelomonocytic progenitor; CM: classical monocytes; IM: intermediate monocytes; NCM: nonclassical monocytes.

Activation of TLR4 × MD − 2 receptor complex by endotoxins results in synthesis of a number of pro- and anti-inflammatory cytokines and chemokines. This is necessary for the rapid and adequate immune response. The synthesized cytokines and chemokines possess both autocrine and paracrine activities, that is why it is hard to reveal certain monocyte subpopulations as main producers of certain cytokines during the developed inflammation. Thus, the model of experimental endotoxemia, a model of primary inflammatory reactions, allows detecting which monocyte subpopulations make the most significant contribution to the synthesis of each cytokine [32, 50] (Figure 3).

The classical monocytes synthesize both pro- and anti-inflammatory cytokines, particularly, TNF*α*, IL-1*β*, and IL-10, in response to LPS stimulation [51].

The subpopulation of intermediate cytokines possessing the signs of both cMo and ncMo expresses significant levels of TNF*α* and IL-10 after LPS stimulation [19]. Besides, CD16+ monocytes are the major producers of IL-6 and IL-8. The levels of these cytokines return to normal values in 24 hours, which points on the absence of the long-term activation of circulating proinflammatory subpopulations of the monocytes [7].

TNF-*α* is produced by all the subtypes of the monocytes, but ncMo is now regarded as its primary producers. Moreover, this subpopulation expresses significant levels of IL-1*β* [19, 33]. The significance of minor CD16+ monocytes is especially high, considering the negative correlation of IL-1*β* levels during sepsis with survival of patients [50]. This shows the dominant role of intermediate and nonclassical monocyte subpopulations during low-grade inflammation in human [7]. Rather, low count of iMo and ncMo in the first moment of LPS activation could limit their contribution to overall level of cytokines and chemokines at this period of time. Nevertheless, one can suppose that these two subpopulations of monocytes regulate levels of TNF-*α*, IL-6, and IL-8 [7, 10]. These data can be useful in development of novel therapeutic approaches to treating sepsis, its progression being linked to elevation of CD16+ monocyte count.

The development of inflammation leads to the formation of tolerance to endotoxins in patients with sepsis. Monocytes of such patients display weak expression of proinflammatory cytokines [52]. Such an effect could be related to elevated number of CD16+ monocytes and activation of the TRIF-dependent pathway “switching” the phenotype of intermediate monocytes from proinflammatory to anti-inflammatory and immunotolerant one [38, 53].

In addition, cytokines synthesized by other cells can influence the activity of monocyte subpopulations. Such a mechanism was shown for interaction of IL-6 with cMo, as well as for interaction of IL-10 with iMo. The authors suggest that elevation of the absolute number of classical monocytes is related to the activation degree of endothelium, the active IL-6 producer. Besides that, elevation of cMo correlates with the severity of sepsis [10].

The effect of IL-10 is a bit different. Under its action, antigen-presenting cells change their phenotype: the relative count of proinflammatory CD16+ decreases, and the portion of CD14+ monocytes performing scavenging function elevates, thus restoring the normal ratio between classical and intermediate monocyte subpopulations. As a rule, such a cycle is optimal for elimination of the pathogen from the blood and prevention of damage of the body by inflammation products [10].

When studying the conditions of cytokine synthesis by monocytes, combination of the results of studies on EE and sepsis allows determining the contribution of each monocyte subpopulation to formation of the cytokine response to inflammatory stimulus and effect of cytokines on the subpopulations themselves. These data can help in developing the targeted therapy effective against the excess of pro-inflammatory cytokines and against immunotolerant condition.

## 8. Conclusions

Subpopulations of monocytes play an essential role in formation of antibacterial defense of the organism. In normal conditions, the changes of absolute and relative counts of the subpopulations, their receptor and cytokine profiles lead to effective elimination of bacteria and their products from the circulation and restoration of homeostasis. During pathological disorders, the changes in monocytes lead to sequential formation of septic condition, hyperinflammation, and immunological tolerance.

Studies on qualitative and quantitative changes of monocyte subpopulations during EE and their comparison with septic monocytes reveal the basic regulatory role of the minor subpopulation of “intermediate” monocytes [7, 8, 31, 33]. These cells possess features of both classical and nonclassical monocytes. The elevated content of intermediate monocytes could be the indicator of acute inflammation [33]. Besides that, the studies of changes of the expression of receptors in monocyte subpopulations during EE and SI can complement each other for more complete understanding of the pattern of progression of this condition.

Alteration of cytokine and receptor profile of monocyte subtypes during septic response development, combined with sepsis markers used up to date (WBC, PCT, CRP, IL-6), can form the bases for early screening and diagnostics of sepsis, which could give rise to use of corresponding therapy soon.

The limitation of the current review is actual equating the systemic inflammation to sepsis. We realize that sepsis is much more complicated process than just inflammation. Nonetheless, a great number of studies of monocytes and their subpopulations was necessary to deepen the understanding of complex pathophysiology of systemic inflammation and sepsis. Thus, use of experimental endotoxemia as a model to study triggering mechanisms of nonregulated inflammatory response could help in development of novel effective approaches to sepsis management.

## Figures and Tables

**Table 1:** Change of the receptor expression on monocytes during EE and SI.

Receptor	Experimental human endotoxemia	SI
TLR2, TLR4	↑(cМО, iMo)	↑ (cMo)
TLR5	↑(iМо)	↑ (cMo, iMo)
CD14/CD16	↓(cMo/ncMo)	↑(iMo,ncMo)
CD11b	↑(cMo)	↑(Mo)
CD64	Not change (Mo)	↑(iMo,ncMo)
CD86	↓(Mo)	↓(Mo)
HLA-DR	↓(iMo, ncMo)	↓(Mo)

↑: increased expression of receptor; ↓: decreased expression of receptor; Mo: monocytes; cMo: classical subpopulation; iMo: intermediate subpopulation; ncMo: nonclassical subpopulation.
